# High‐Energy LiNiO_2_ Li Metal Batteries Enabled by Hybrid Electrolyte Consisting of Ionic Liquid and Weakly Solvating Fluorinated Ether

**DOI:** 10.1002/advs.202409662

**Published:** 2024-10-17

**Authors:** Qian Liu, Jiayi Xu, Wei Jiang, Jihyeon Gim, Adam P. Tornheim, Rajesh Pathak, Qijia Zhu, Peng Zuo, Zhenzhen Yang, Krzysztof Z. Pupek, Eungje Lee, Chongmin Wang, Cong Liu, Jason R. Croy, Kang Xu, Zhengcheng Zhang

**Affiliations:** ^1^ Chemical Sciences and Engineering Division Argonne National Laboratory 9700 S. Cass Ave. Lemont IL 60439 USA; ^2^ Computational Science Division Argonne National Laboratory 9700 S. Cass Ave. Lemont IL 60439 USA; ^3^ Applied Material Division Argonne National Laboratory 9700 S. Cass Ave. Lemont IL 60439 USA; ^4^ Environmental Molecular Sciences Laboratory Pacific Northwest National Laboratory Richland WA 99352 USA; ^5^ Battery Science Branch Energy Science Division Sensor and Electron Devices Directorate U.S. Army Research Laboratory Adelphi MD 20783 USA

**Keywords:** ionic liquid, Li metal, LiNiO_2_, MD simulation

## Abstract

In pursuit of the highest possible energy density, researchers shift their focus to the ultimate anode material, lithium metal (Li^0^), and high‐capacity cathode materials with high nickel content (Ni > 80%). The combination of these aggressive electrodes presents unprecedented challenges to the electrolyte. Here, we report a hybrid electrolyte consisting of a highly fluorinated ionic liquid and a weakly solvating fluorinated ether, whose hybridization structure enables the reversible operation of a battery chemistry based on Li^0^ and LiNiO_2_ (Ni = 100%), delivering nearly theoretical capacity of the latter (up to 249 mAh g^−1^) for >300 cycles with retention of 78.6% and in absence of unwanted morphological changes in both electrodes. Extensive characterization assisted by molecular dynamic simulation and density functional theory calculations reveals the function of the fluorinated ether to be far more profound than simple dilution and viscosity reduction. Instead, it induces drastic changes in Li^+^‐solvation environment, the consequence of which engenders simultaneous stabilization of electrode/electrolyte and interfacing via formation of respective interfacial chemistries. This study further unlocks fundamental knowledge underneath the prevailing “diluent strategy” that is extensively applied by the electrolyte researchers and opens more design space for the next‐generation electrolytes and interphases for these coveted battery chemistries.

## Graphical abstract

A hybrid electrolyte consisting of a highly fluorinated ionic liquid and a weakly solvating fluorinated ether induces drastic changes in Li^+^‐solvation environment, the consequence of which engenders simultaneous stabilization of Li^0^/electrolyte and LiNiO2/electrolyte interfacing via formation of respective interfacial chemistries.

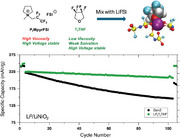

## 1 Introduction

Following the successful commercialization of lithium‐ion batteries (LIBs) and the electrification revolution in our daily life it caused, the mankind now actively pursues the ambitious goal of decarbonizing our entire economy and civilization in the coming decades, which requires batteries of higher energy density, besides excellent reversibility (cycle‐life and calendar life), low cost, earth abundance as well as safety.^[^ ^1^, ^2^ ^]^ Thermodynamically speaking, lithium‐metal (Li^0^) remains the irreplaceable “ultimate anode material” because it possessssses unique properties (the lowest electrode potential, −3.04 V vs standard hydrogen reference, and the second highest specific capacity, 3856 mAh g^−1^) that almost no other elements on the Periodic Table could compete.^[^ ^3^ ^]^ Since the late 1960s researchers have been pursuing a rechargeable lithium‐metal battery as the “Holy Grail” with little success.^[^ ^4^ ^]^ On the other side of the battery, several high‐capacity cathode materials present varying opportunities, which range from conversion‐reaction chemistries (metal fluorides, sulfides, and oxygen) that remain in experimental stages, to intercalation hosts that closely simulate the cathode materials used in the mature LIBs but with modifications in both chemical composition and lattice structures.^[^ ^5^ ^]^ Among these, the layer‐structured transition metal oxides with high nickel (Ni) contents are apparently more promising and practical, and lithium nickel manganese cobalt oxides with Ni content above 80% (LiNi_0.8_Mn_0.1_Co_0.1_O_2_, or NMC811) have been successfully adopted in LIBs for the new generation of electric vehicles. Driven by the desire of higher capacity, which is directly proportional to Ni content and the upper voltage the cell is charged to, and the desire to reduce the reliance on Co, which is a rare element with both geopolitical and ethical risks, a layer‐structured transition metal oxide with 100% Ni content, i.e., LiNiO_2_ (LNO) with theoretical specific capacity of 275 mAh g^−1^, seems to be the ideal and natural candidate.

However, these two powerful electrode materials, Li^0^ and LNO, also come with their issues, with the former known for its reactivity with all known electrolyte materials and its natural tendency of forming dangerous morphologies (dendritic and dead Li^0^), and the latter for its structural instability induced by the high reactivity of Ni^4+^ species, and the subsequent loss of oxygen from its lattice leading to catastrophic consequences. The combination of the two presents unprecedented challenges to the electrolytes, which must stabilize both electrode materials simultaneously.^[^ ^6^, ^7^, ^8^, ^9^, ^10^, ^11^, ^12^, ^13^, ^14^ ^]^ Previously, Manthiram and co‐workers have demonstrated that, by resorting to the so‐called “locally concentrated electrolyte” and fluorinated esters and ethers, it is possible to harness the instability and reactivity of LNO, making the Li^0^/LNO battery reversible.^[^ ^15^, ^16^ ^]^ In this work, we further explored electrolyte engineering as a feasible pathway to enable the powerful Li^0^/LNO chemistry via the hybridization of two completely different electrolyte systems, i.e., fluorinated ionic liquid and fluorinated ether, and attempted to shed light on the fundamental mechanisms underneath.

In general, ionic liquids (ILs) consisting of pyrrolidinium‐based cations and fluorinated sulfonyl imide anions, either bis(fluorosulfonyl)imide (FSI^−^) or bis(trifluoromethanesulfonyl)imide (TFSI^−^), have high stability against both reduction and oxidation by forming protective interphases on both electrodes.^[^ ^17^, ^18^, ^19^, ^20^, ^21^, ^22^, ^23^, ^24^ ^]^ Our previous work further revealed that by substituting fluorine on the organic cation structure, one could significantly alter the inner‐Helmholtz interfacial structure near Li^0^ surface, thus introducing more fluorine content into the eventual interphases.^[^ ^25^ ^]^ While a well‐established design philosophy of interphases indicates that highly fluorinated interphases provide better protection, fluorination does induce higher viscosity and lower ion transport. On the other hand, it has also been well‐established that dilution of an ionic liquid with ethers^[^ ^26^, ^27^, ^28^, ^29^, ^30^, ^31^, ^32^ ^]^ or functionalizing an organic cation with ether linkage, works in the opposite direction, i.e., effectively reducing viscosity and improving conductivity but in the expense of high voltage stability.^[^ ^33^, ^34^, ^35^, ^36^, ^37^, ^38^, ^39^, ^40^ ^]^

Based on the above knowledge, we designed and synthesized a highly fluorinated ionic liquid, 1‐methyl‐1‐(2,2,3,3,3‐pentafluoropropyl)pyrrolidinium bis(fluorosulfonyl)imide (P_f_MpyrFSI), for the challenging chemistry of Li^0^/LNO. The poly‐fluorinated cation is expected to suppress the reactivity with both cathode and anode with respective fluorinated interphases but would also substantially increase the viscosity. To mitigate such compromises, fluorinated ether diluents, 1,1,2,2‐tetrafluoroethyl 2,2,3,3‐tetrafluoropropyl ether (TTE), and 3,3,4,4‐Tetrafluorotetrahydrofuran (T_f_THF), were selected as “diluents” due to their high voltage stability and non/weak solvating capability. Extensive characterizations were conducted to reveal the liquid structure of the hybrid electrolyte consisting of the fluorinated ionic liquid and the fluorinated ethers, the subsequent interfacial chemistries and corresponding cell performance. The weakly solvating T_f_THF presents the best performance, enabling stable cycling of Li^0^/LNO battery up to 4.3 V with average specific capacity of 220 mAh g^−1^ for 300 cycles, which can be directly correlated to its preferential reduction on Li^0^ surface due to its cyclic structure and the subsequent binding capability with Li^+^.

## 2 Results and Discussion

### 2.1 Hybridizing Fluorinated Ionic Liquid with Fluorinated Ether

**Figure 1:**
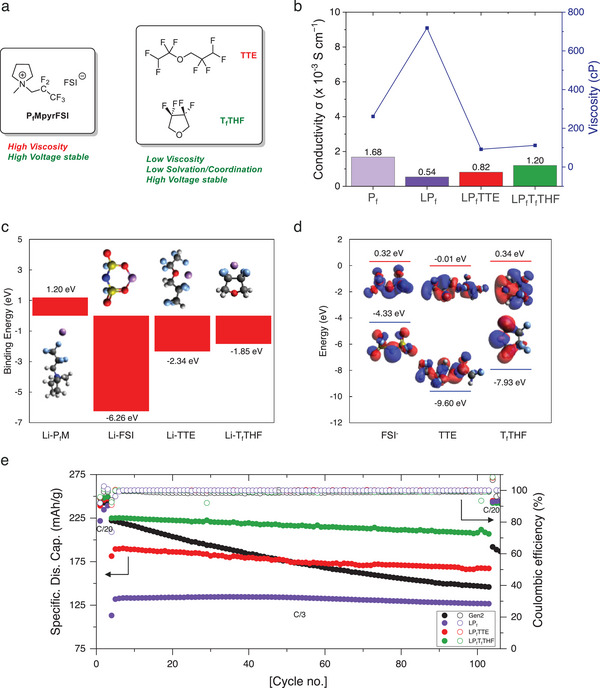
a) Structure of P_f_MpyrFSI, TTE and T_f_THF. b) Viscosity and conductivity of P_f_MpyrFSI, LP_f_, LP_f_TTE, and LP_f_T_f_THF, c) Li^+^ binding energies of P_f_M, FSI^−^, TTE, and T_f_THF. d) HOMO‐LUMO energy levels of FSI^−^, TTE, and T_f_THF. e) LiNiO_2_/Li cycling performance using Gen2, LP_f_, LP_f_TTE and LP_f_T_f_THF electrolytes with cutoff voltage of 4.3–3.0 V at 30 °C.

P_f_MpyrFSI was synthesized using the one‐step quaternization method following literature procedure.^[^ ^41^ ^]^ As expected, the highly fluorinated side chain of P_f_MpyrFSI brings a high viscosity of 261 cP (**Figure** **1**a) along with a wide electrochemical window and high anodic stability up to 5.5 V versus Li^+^/Li (Figure S2, Supporting Information). After dissolving lithium bis(fluorosulfonyl)imide (LiFSI) salt in P_f_MpyrFSI by 2:3 molar ratio, the electrolyte [LiFSI]_2_[P_f_MpyrFSI]_3_ (LP_f_) shows an even higher viscosity of 718 cP, with a conductivity of only 0.54 × 10^−3^ S cm^−1^. Such slow ion transport would prevent its application in batteries, when the typical viscosity and ion conductivity of LIB electrolytes usually ranges ≈4 cP and 10 × 10^−3^ S cm^−1^, respectively. Fluorinated ethers, TTE and T_f_THF are selected as diluent for LP_f_ electrolyte, because they all possess the merits of: 1) low viscosity, 2) low coordination/solvation capability with Li^+^, and 3) high voltage stability. Based on the density functional theory (DFT) calculation, the binding energies of the fluorinated ethers to Li^+^ ion are much weaker compared to Li^+^‐FSI^−^ binding energy of −6.26 eV (Figure 1c), which implies strong Li^+^‐anion cluster formation in the hybrid electrolyte. The calculated highest occupied molecular orbital (HOMO)‐lowest unoccupied molecular orbital (LUMO) energy levels also show T_f_THF has higher HOMO level (−7.93 eV) compared to TTE (−9.60 eV), which are both lower than the HOMO level of the fluorinated ionic liquid P_f_MpyrFSI (−7.86 eV) (Figure 1d; Figure S3, Supporting Information), suggesting the fluorinated ethers both have good stability against oxidation. The hybrid electrolytes are prepared by mixing LiFSI/P_f_MpyrFSI/diluent at 2/3/3 molar ratio. [LiFSI]_2_[P_f_MpyrFSI]_3_[TTE]_3_ (LP_f_TTE) and [LiFSI]_2_[P_f_MpyrFSI]_3_[T_f_THF]_3_ (LP_f_T_f_THF) both show a substantially lower viscosity of 92 and 117 cP, respectively. The corresponding conductivities of these electrolytes are 0.82 × 10^−3^ S cm^−1^ for LP_f_TTE and 1.20 × 10^−3^ S cm^−1^ for LP_f_T_f_THF. The ethers also improve the wettability of the electrolyte as shown in Figure S4 (Supporting Information), where the contact angle decreases to 41°–42° for LP_f_ electrolyte.

The initial electrochemical tests of the ionic liquid and its hybrid electrolytes are conducted with the high‐capacity LiNiO_2_ cathode paired with an excess Li^0^ anode (125 µm) that is cycled between 4.3 and 3.0 V. The state‐of‐the‐art carbonate electrolyte Gen2 serves as a comparison (Figure 1e). LiNiO_2_ in Gen2 delivers a high initial discharge capacity of 240.6 mAh g^−1^ at C/20 and 222.2 mAh g^−1^ at C/3. However, due to the instability in the Li^0^/LNO couple, the capacity rapidly drops to 145.9 mAh g^−1^ at C/3 after 100 cycles and 186.8 mAh g^−1^ at C/20. For the neat ionic liquid, its high viscosity makes the wetting process sluggish, as indicated by the gradually increasing capacity during the first 3 C/20 cycles. At the third C/20 cycle, 239.3 mAh g^−1^ capacity is finally reached in the ionic liquid, comparable to Gen2 electrolyte. However, once the C‐rate increases to C/3, the discharge capacity is substantially lowered to only 131.9 mAh g^−1^. Only 5 mAh g^−1^ capacity loss (≈2%) at C/3 was observed after 100 cycles, and a full capacity recovery of 244.7 mAh g^−1^ occurred at C/20. The cycling results suggest that LP_f_ electrolyte possess intrinsic stability in the electrochemical environment created by Li^0^/LNO couple, but its high viscosity constitutes a kinetic barrier for normal battery operation. With the TTE as diluent, the hybrid LP_f_TTE also shows an initial wetting process but starts from a much higher capacity of 239.2 mAh g^−1^ and reaches 247.3 mAh g^−1^ at the third C/20 cycle. When increasing the C‐rate to C/3, the discharge capacity is decreased to 189.4 mAh g^−1^, which is lower than Gen2 electrolyte. Although TTE‐presence improves the rate capability, the capacity retention is only 88% at C/3 over 100 cycles, implying a negative impact of TTE on stability. The hybrid electrolyte LP_f_T_f_THF shows a similar initial C/20 performance as LP_f_TTE, delivering 249.4 mAh g^−1^ discharge capacity at the third C/20 cycle. This number is over 90% of the theoretical capacity of LNO (275 mAh g^−1^). When tested at C/3 rate, LNO in LP_f_T_f_THF electrolyte delivers a high initial discharge capacity of 224.9 mAh g^−1^, which is comparable to Gen2 electrolyte and much higher than LP_f_ and LP_f_TTE electrolytes. The capacity retention of LNO in LP_f_T_f_THF shows the highest retention of 92% at C/3 over 100 cycles. Therefore, although both TTE and T_f_THF substantially lower the electrolyte viscosity and meet the selection criteria of high voltage stability, their eventual behavior in the Li^0^/LNO couple vary, where T_f_THF shows better rate capability improvement and minimum impact on the electrolyte cyclability.

### 2.2 Li^+^ Solvation Environment Through MD Simulations

**Figure 2:**
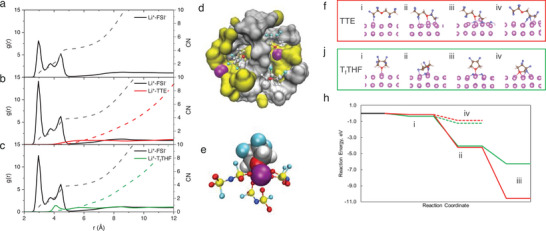
MD simulation of bulk electrolyte structures. Radial distribution functions (RDFs) and coordination numbers of FSI^−^ anion and diluents for a) LP_f_, b) LP_f_TTE, and c) LP_f_T_f_THF electrolytes. d) Snapshot of MD simulated box of LP_f_T_f_THF electrolyte: silver clouds, yellow clouds, and purple spheres represent P_f_Mpyr^+^ cation, FSI^−^ anion and Li^+^ ion, respectively. T_f_THF is shown in ball‐stick model: C‐gray, H‐white, O‐red, and F‐cyan. e) A representative Li^+^ ion solvation structure with T_f_THF participation: C‐gray, H‐white, O‐red, N‐blue, S‐yellow, and F‐cyan. DFT calculation of optimized Structures of reaction intermediates of f) TTE and j) T_f_THF on Li metal surface, and h) potential energy surface of the proposed reaction mechanism: defluorination (solid line) and deprotonation (dashed line).

To reveal what leads to the performance difference among different fluorinated ethers, molecular dynamics simulation (MD) is conducted to further understand the bulk electrolyte structure. Li^+^ solvation structure is characterized through radial distribution functions (RDF) (**Figure** **2**a–c; Figure S5, Supporting Information). In neat ionic liquid, Li^+^ is solely solvated by FSI^−^ as suggested by the strong correlation of Li^+^‐FSI^−^ with a sharp peak at 2.95 Å and negligible intensity of Li^+^‐cation below 5 Å. For hybrid electrolyte LP_f_TTE, similar Li^+^ solvation structures dominated by FSI^−^ are observed: TTE shows a negligible correlation with Li^+^. These results are also consistent with the weak Li^+^ ion binding energy with TTE calculated by DFT. Interestingly, T_f_THF shows a weaker correlation with Li^+^ as a small peak observed at 4.05 Å, suggesting LP_f_T_f_THF adopts a slightly different Li^+^ solvation structure, where FSI^−^ still dominates but T_f_THF participates to a certain extent. The Li^+^ should be solvated by 3.90 FSI^−^ and 0.5 T_f_THF, respectively. Figure 2d shows a snapshot of LP_f_T_f_THF, in which silver clouds, yellow clouds, and purple spheres represent FSI^−^, cation, and Li^+^ ion, respectively, and T_f_THF is shown in the ball‐and‐stick model. In this picture, Li^+^ is embedded in the FSI^−^ cloud and only one T_f_THF is coordinated with Li^+^ through the O center, and the other two T_f_THF molecules are free. This result is in contrast with the Li^+^ binding energy from the DFT calculation, where T_f_THF shows a lower Li^+^ ion binding capability compared to TTE and interacts with Li^+^ through the F centers, which could be due to the gas phase model used in this DFT calculation. Figure 2e shows a representative Li^+^ solvation structure with the participation of T_f_THF. The different Li^+^ solvation capability of T_f_THF from TTE is ascribed to the molecular structure. TTE is linear fluorinated ethers, whose flexibility in conformational conversion reduces the molecular polarity; while the ring‐structure of T_f_THF essentially forbids any conformational conversion, thus making the oxygen center more sterically accessible to the incoming Li^+^, leading to higher polarity of T_f_THF than TTE. In addition to the Li^+^ ion solvation capability difference, the fluorinated ethers also impact the FSI^−^ anion and organic cation distribution. With the addition of TTE, the FSI^−^ anion shows the highest correlation peak intensity with Li^+^ ion, suggesting a stronger Li^+^‐FSI^−^ interaction, which is consistent with the ^7^Li NMR and ^19^F NMR results that for LP_f_TTE electrolyte, Li^+^ resonance is downfield shifted and F resonance in FSI is upfield shifted compared to the LP_f_ (Figure S5e,f, Supporting Information). Moreover, as shown in Figure S5a (Supporting Information) pyrrolidinium shows a higher peak intensity ≈5 Å in LP_f_TTE, while LP_f_T_f_THF shows a similar cation trace as LP_f_ electrolyte. This result suggests that although TTE does not directly participate in Li^+^ solvation, it pushes the pyrrolidinium cation slightly closer to the Li^+^, which disturbs the Coulombic field of Li^+^. Along with the strong Li^+^‐FSI^−^ interaction, this could potentially lead to sluggish ion transport. The MD result agrees with the observation in ^1^H NMR and ^19^F NMR (Figure S5d–g, Supporting Information), that cation resonances in LP_f_TTE all have shown down‐field shift in ^1^H NMR compared to neat ionic liquid and LP_f_T_f_THF, indicating a slightly electron deficient chemical environment, while resonances in ^19^F NMR show an upfield shift. Compared to TTE, although T_f_THF participates in Li^+^ solvation, as indicated by the MD results and similar chemical shifts in the NMR spectra compared to LP_f_ electrolyte (Figure S5, Supporting Information), interestingly it does not substantially disturb other ions distribution and maintains most of the bulk electrolyte structure in LP_f_ electrolyte.

To further understand how the presence of fluorinated ethers is related to battery performance, electrolyte decompositions over Li (110) surface were studied, so that the interphases could be brought in as the intermediate factor. The potential energy surfaces, Figure 2f–h and Figure S6 (Supporting Information) were constructed based on DFT calculation to elucidate the reaction mechanism for SEI formation. By introducing the fluorinated ethers to Li (110) surface, TTE binds to Li^0^ surface via F atoms (Figure 2f), while T_f_THF binds to Li^0^ surface via O (Figure 2j). Intuitively, both fluorinated ethers are expected to bind via O atom, but the flexible molecular structures of TTE sterically hinder the O‐site, therefore only leaving F to interact with Li^0^. On the other hand, the cyclic structure of T_f_THF exposes O‐site, which strongly binds to Li^0^ with −0.35 eV binding energy, compared to −0.14 eV for TTE. The decomposition of these fluorinated ether was also considered via both deprotonation and defluorination, the two most probable reaction pathways. As shown in Figure 2h, both TTE and T_f_THF thermodynamically favor the defluorination pathway (Figure 2h‐iii) over deprotonation (Figure 2h‐iv), resulting in simultaneous dissociation of three or two C─F bonds, respectively. Defluorination of TTE is more exothermic (−10.57 eV) compared to that of T_f_THF (−6.27 eV), suggesting TTE could have higher contribution to the SEI formation. FSI^−^ anion is another key contributor to interfacial chemistry. To reveal how FSI^−^ is activated under the influence of fluorinated ethers, a S─F bond of FSI^−^ dissociation was calculated, whose reaction energy in presence of TTE is −6.05 eV (Figure S6a‐iii, Supporting Information), a value slightly less exothermic than the S–F dissociation in presence of T_f_THF (−6.43 eV, Figure S6b‐iii, Supporting Information). To break the second S─F bond, under the influence of TTE and T_f_THF, the reaction energies are also exothermic by −5.84 and −5.33 eV, respectively (Figure S6a‐iv,b‐iv, Supporting Information). Overall, the reaction profile of LiFSI decomposition with co‐adsorption of TTE and T_f_THF is fairly comparable. Based on the above results, we anticipate that TTE is more susceptible to reductive decompositions than T_f_THF and both fluorinate ethers will lead to a LiF‐rich SEI due to the thermodynamic favored defluorination pathway.

### 2.3 Interfacial Chemistries

**Figure 3:**
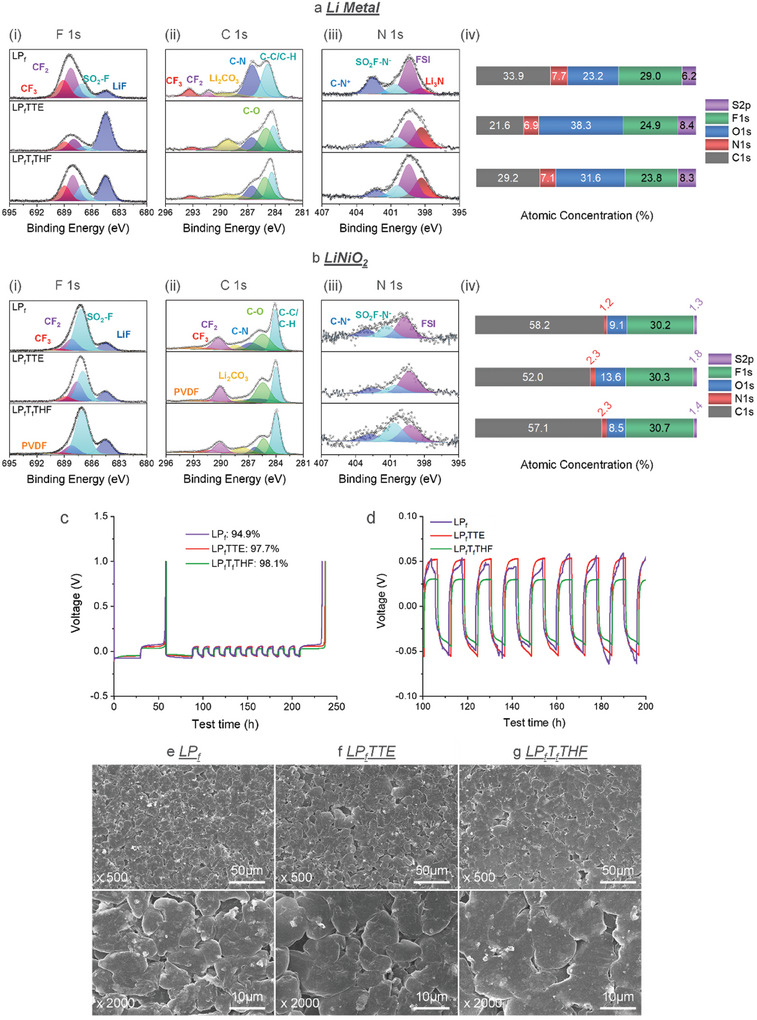
XPS analysis of recovered a) Li metal electrodes and b) LiNiO_2_ cathodes: (i) F 1s spectra, (ii) C 1s spectra, (iii) N 1s spectra, and (iv) atomic concentration using LP_f_, LP_f_TTE, and LP_f_T_f_THF electrolytes. Cu/Li cell performance of an Aurbach test c) and expanded voltage profiles d) using LP_f_, LP_f_TTE, and LP_f_T_f_THF electrolytes with a current density of 0.1 mA cm^−2^. SEM of plated Li on Cu foil with a current density of 0.1 mA cm^−2^ using e) LP_f_, f) LP_f_TTE, and g) LP_f_T_f_THF electrolytes.

The interfacial chemistries for the neat ionic liquid and its hybridized electrolytes were studied by analyzing solid‐electrolyte‐interphase (SEI) on Li^0^ anode and cathode‐electrolyte‐interphase (CEI) on LNO cathode using X‐ray photoelectron spectroscopy (XPS, **Figure** **3**). The Li^0^ and LNO samples are harvested after 3 formation cycles at C/20 rate, whose XPS results are summarized in Figure 3a,b. On Li^0^ surface, four F‐containing components are observed in F 1s spectra for neat ionic liquid, which are LiF at 684.5 eV, ─SO_2_F at 687.1 eV, ─CF_2_ at 688.3 eV and ─CF_3_ at 689.1 eV. The ─CF_2_ and ─CF_3_ species originated from the decomposition of P_f_Mpyr^+^ cation and ─SO_2_F species from the decomposition of FSI^−^ anion. In neat ionic liquid, P_f_Mpyr^+^ cation is the sole source of carbon atoms, therefore C 1s spectra represent this cation decomposition, where signature peaks C–N, ─CF_2_, and ─CF_3_ are observed at 286.5, 291.3, and 293.4 eV, respectively. For N 1s spectra, the peak observed at 402.6 eV is associated with cation decomposition, and the other three peaks observed at 400.7, 399.4, and 398.4 eV are associated with different degrees of FSI^−^ anion decomposition, where the peak at 398.4 eV represents the fully reduced product and is assigned to be Li_3_N. Both P_f_Mpyr^+^ cation and FSI^−^ anion contributes to the SEI formation in the neat ionic liquid. When fluorinated ethers are introduced, the main SEI component bears a close resemblance to that from neat ionic liquid, but the quantitative ratio of each component is altered. For LP_f_TTE electrolytes, a high LiF content is observed in F 1s spectra, which is assigned to the decomposition of TTE. As suggested by DFT calculation, TTE favors a defluorination pathway involving multiple C─F bonds dissociation simultaneously, making LiF the dominant component. This relative LiF content is the highest among the three electrolytes. Another TTE decomposition peak is observed at 285.0 eV in C 1s spectra and assigned to be C–O peak. TTE also impacts the relative contributions from P_f_Mpyr^+^ cation and FSI^−^ anion to the SEI. In N 1s spectra, the relative peak intensity at 402.5 eV is much reduced compared to what is observed in neat ionic liquid, suggesting that less P_f_Mpyr^+^ cation decomposed with the addition of TTE. Moreover, the Li_3_N relative peak intensity at 398.3 eV is also increased, indicating the addition of TTE promotes a more complete reduction of FSI^−^ anion. Similar impacts on the SEI composition are observed for T_f_THF, but the extent varies. For LP_f_T_f_THF, a relatively high LiF content is observed in F 1s spectra compared to neat ionic liquid, but much less when compared to LP_f_TTE. Reduced P_f_Mpyr^+^ cation decomposition peak intensity and higher Li_3_N peak intensity are also observed with the addition of T_f_THF. The atomic concentrations further shed some light on the contribution of each component in the electrolyte (Figure 3a‐iv). As the S atom can only be contributed by FSI^−^ anion decomposition and the O atom could come from both FSI^−^ anion and fluorinated ethers, O atomic concentrations are consequently higher for all hybrid electrolytes while S atomic concentrations only slightly varies from neat ionic liquid, which reflects the actual contribution from fluorinated ethers to SEI. LP_f_TTE electrolyte shows higher O atomic concentrations compared to LP_f_T_f_THF, suggesting that TTE is more readily reduced on Li^0^ when compared to T_f_THF. This result agrees with the DFT calculation, where TTE prefers to decompose on Li^0^ via defluorination pathway and is more exothermic compared to T_f_THF.

On the LNO surface, the decomposition species of neat ionic liquid and hybridized electrolytes are very similar to what observed on Li^0^, except that Li_3_N peaks are absent because it is the fully reduced product of FSI^−^ anion. Since the decomposition species on the cathode surface are mainly derived from oxidative reactions of electrolyte components, the concentrations of the components are altered. As shown in Figure 3b‐iv, the atomic concentrations of N and S are very small, suggesting that only a small amount of P_f_Mpyr^+^ cation and FSI^−^ anion are oxidized on LNO surface, which is consistent with its high oxidation stability as indicated by cyclic voltammetry results and HOMO energy level from the DFT calculation. Moreover, the M–O peak of the cathode bulk can be observed at 528.6 eV in O 1s spectra (Figure S7, Supporting Information) with neat ionic liquid, suggesting a thin CEI layer. With TTE present, O atom concentration increases to 13.6% from 9.1%, which could be contributed by TTE oxidation. The relative M–O peak intensities are also obscured by the thicker CEI formed in the presence of this fluorinated ether. For LP_f_T_f_THF electrolyte, the O atomic concentration is only 8.5% and comparable to neat ionic liquid. The M‐O peak relative intensity is also similar to what is observed in neat ionic liquid, suggesting that T_f_THF behaves differently from TTE, and LP_f_T_f_THF electrolyte forms a thin CEI layer as the neat ionic liquid does. Although T_f_THF itself is the most prone to oxidation among the three fluorinated ethers due to the highest HOMO energy level (−7.93 eV), thanks to its Li^+^ coordination capabilities, the T_f_THF in Li^+^ solvation sheath is more resistive to oxidation, as indicated by the HOMO energy level at −12.4 eV (Figure S3, Supporting Information), which leads to a thin CEI formation.

### 2.4 Li^0^ Reversibility and Surface Morphology

Li^0^ compatibility with ionic liquid and the three fluorinated ethers are examined by evaluating Coulombic efficiency (CE) of Cu/Li cells with Aurbach CE Protocol^[^ ^42^ ^]^ and investigating the Li morphology via scanning electron microscopy (SEM) (Figure 3e–h). Neat ionic liquid shows a Coulombic efficiency of 94.9%. Due to its high viscosity, the voltage profile exhibits an “arcing” behavior corresponding to a diffusion‐controlled process, and a high overpotential of 50 mV is observed. The deposited Li^0^ adopts a densely packed columnar morphology (Figure 3e). Upon addition of fluorinated ethers, the Coulombic efficiency for LP_f_TTE and LP_f_T_f_THF are increased to 97.7% and 98.1%, respectively. Interestingly, with TTE, although the viscosity of the bulk electrolyte is substantially reduced, the cell overpotential is not reduced accordingly but remains at ≈50 mV. This result could be attributed to the high LiF content formed by TTE on Li^0^. LiF has been considered a critical SEI component that protects Li^0^ from extensive electrolyte decomposition,^[^ ^43^, ^44^, ^45^, ^46^ ^]^ however, LiF is also known to be an ionic insulator.^[^ ^47^, ^48^, ^49^ ^]^ Therefore, the concentration of LiF in SEI must be balanced to enable reversible Li^0^ deposition/stripping without inducing high overpotential. For LP_f_TTE electrolyte, the high overpotential on Li metal also results in the lower rate capability of Li^0^/LNO cell and lower discharge capacity at C/3 rate as observed in Figure 1. With T_f_THF, the LiF concentration in the SEI is higher than what is observed in LP_f_ electrolyte, but much lower than LP_f_TTE electrolyte. These balanced LiF concentrations result in lower cell overpotentials observed with LP_f_T_f_THF electrolyte (30 mV) in Cu/Li cells, which lead to the higher rate capability and higher capacity at C/3 in Li^0^/LNO cells. With addition of the fluorinated ether diluents, the deposited Li^0^ morphology becomes denser with larger particles, especially for T_f_THF. XRD analysis of cycled Li metal also shows LP_f_T_f_HF promotes the generation of the more stable crystalline plane (110) Li formation (Figure S9, Supporting Information).^[^ ^50^ ^]^ Overall, the fluorinated ethers improve Li^0^ compatibility with electrolyte, where T_f_THF shows better performance due to the formation of SEI with balanced LiF content.

### 2.5 Li^0^/LNO Battery

**Figure 4:**
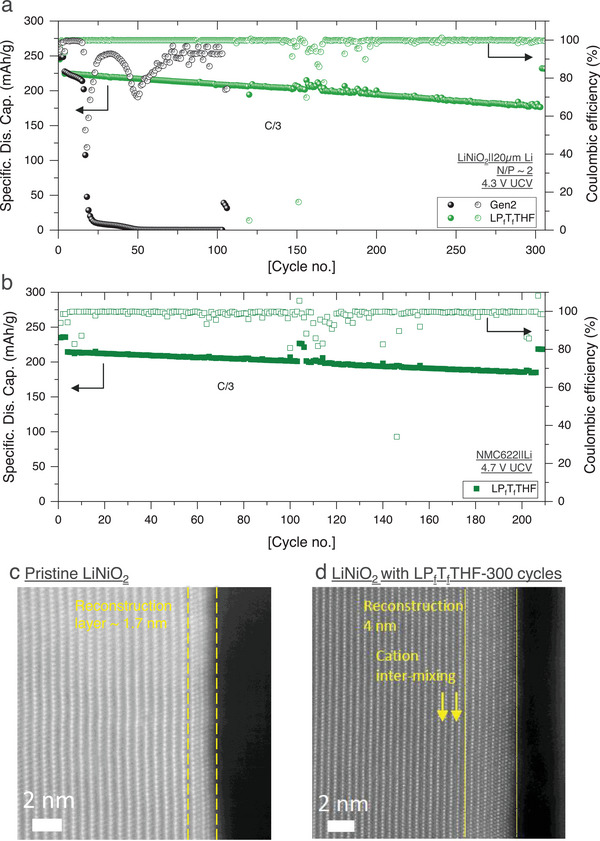
a) Long‐term LiNiO_2_/Li‐20 µm cycling performance using Gen2 and LP_f_T_f_THF electrolytes with a cutoff voltage of 4.3–3.0 V at 30 °C. b) Cycling performance of NMC622/Li cell using LP_f_T_f_THF electrolyte with a cutoff voltage of 4.7–3.0 V at 30 °C. Note: Some fluctuation of the CE in the middle of the cell testing is observed due to the temperature chamber control issue. HAADF‐STEM analysis of LiNiO_2_ cathodes: c) pristine LiNiO_2_ cathode and d) cycled LiNiO_2_ cathode with LP_f_T_f_THF electrolyte after 300 cycles.

Based on the initial Li^0^/LNO cell performance, bulk electrolyte structure, and surface chemistry analysis, as well as Li^0^ morphology and reversibility, LP_f_T_f_THF electrolyte is selected to be further evaluated for long‐term cycling performance with limited Li^0^ inventory (20 µm). Gen2 electrolyte is also tested under the same condition as a comparison. The cycling results are summarized in **Figure** **4**. Due to the poor compatibility with Li^0^ and limited Li inventory, Gen2 electrolyte shows a rapid capacity decay within 16 cycles. In comparison, a stable cycling performance over 300 cycles is achieved in LP_f_T_f_THF electrolyte, with 78.6% capacity retention at C/3 rate. At C/20 rate, LP_f_T_f_THF electrolyte can deliver 231.5 mAh g^−1^ capacity at cycle 306. This excellent cycling performance is attributed to the intrinsic stability of fluorinated ionic liquid P_f_MpyrFSI and the cyclic structure of T_f_THF with weak solvation capability for Li^+^ that promotes the desired interfacial chemistries as elucidated by the XPS analysis. High‐resolution transmission electron microscopy (STEM) reveals that the LNO surface is well preserved in LP_f_T_f_THF electrolyte after extended cycling. On a pristine LNO surface, a 1.7 nm reconstruction layer is visible, which only increased to 4 nm after 300 cycles. This TEM result confirms that LP_f_T_f_THF electrolyte can effectively minimize the degradation of the layer structure due to intrinsic lattice stress induced by the Ni^4+^ cations. The high voltage stability of the LP_f_T_f_THF electrolyte is also demonstrated by its performance on an alternative high nickel cathode material, NMC622, where an 86.4% capacity retention is achieved at cycle 200 with an upper cutoff voltage of 4.7 V, delivering ≈210 mAh g^−1^ capacity (Figure 4b).

## 3 Conclusion

A hybrid electrolyte based on highly fluorinated ionic liquid and fluorinated ethers was explored for the aggressive Li^0^/LNO battery chemistry. Between the two fluorinated ethers investigated, T_f_THF demonstrates excellent cell performance due to its capability of weakly solvating Li^+^ and forming protective SEI and CEI simultaneously. MD simulation reveals that the cyclic structure of T_f_THF dictates such unique behaviors. The fundamental understanding of the prevailing “diluent strategy” opens more design space for the next‐generation electrolytes and interphases for these coveted battery chemistries.

## Conflict of Interest

The authors declare no conflict of interest.

## Supporting information



Supporting Information

## Data Availability

The data that support the findings of this study are available in the supplementary material of this article.
