# Supplementary material for: High‐Energy LiNiO2 Li Metal Batteries Enabled by Hybrid Electrolyte Consisting of Ionic Liquid and Weakly Solvating Fluorinated Ether
Source: Adv Sci (Weinh). 2024 Oct 17;11(46):2409662. doi: 10.1002/advs.202409662 (PMC11633521; doi:10.1002/advs.202409662)
Supplement: Supplementary file 1 — Supporting Information [file ADVS-11-2409662-s001.docx]

Supplementary Information

**High-Energy LiNiO_2_ Li Metal Batteries Enabled by Hybrid Electrolyte Consisting of Ionic Liquid and Weakly Solvating Fluorinated Ether**

Qian Liu^1^, Jiayi Xu^1^, Wei Jiang^2^, Jihyeon Gim^1^, Adam P. Tornheim^1^, Rajesh Pathak^3^, Qijia Zhu^1^, Peng Zuo^4^, Zhenzhen Yang^1^, Krzysztof Z. Pupek^3^, Eungje Lee^1^, Chongmin Wang^4^, Cong Liu^1^, Jason R. Cory^1*^, Kang Xu^5*^, and Zhengcheng Zhang^1*^

^1^Chemical Sciences and Engineering Division

^2^Computational Science Division

^3^Applied Material Division

Argonne National Laboratory

9700 S. Cass Ave., Argonne, IL 60439

^4^Environmental Molecular Sciences Laboratory, Pacific Northwest National Laboratory, Richland, Washington 99352

^5^Battery Science Branch, Energy Science Division, Sensor and Electron Devices Directorate, US Army Research Laboratory, Adelphi, MD, USA

^*^Email: croy@anl.gov; conrad.k.xu.civ@army.mil; zzhang@anl.gov

***Electrode and electrolyte preparation.*** LiNiO_2_ cathode (90% LiNiO_2_; 5% C45 conductive carbon, Timcal; 5% PVdF, Solvay 5130) was fabricated by the CAMP (Cell Analysis Modeling and Prototyping) Facility at Argonne National Laboratory as part of an effort from a DOE (Department of Energy)-VTO (Vehicle Technologies Office) funded consortium project RNGC (Realizing Next Generation Cathode). Active material loading is 6.6 mg/cm^2^. 1-Methyl-1-​(2,​2,​3,​3,​3-​pentafluoropropyl)​pyrrolidinium bis(fluorosulfonyl)imide (P_f_MpyrFSI) was synthesized following the literature procedure^1^. The resultant ionic liquid was dried in a lyophilizer for at least two days, then stored in 4 Å molecular sieves and filtered before use. The water content was <20 ppm, measured by Karl-Fischer titrator C30. Electrolytes were prepared by dissolving LiFSI (Nippon ShokuBai Co., Ltd.) in P_f_MpyrFSI w/o diluents in an argon-filled glovebox.

***Physical and electrochemical properties.*** Galvanostatic charge-discharge cycling tests were conducted on the Maccor Electrochemical Analyzer (MIMSclient) with Al-coated 2032-coin cells. LiNiO_2_/Li cells were tested with a cutoff voltage of 4.3-3.0 V and NMC622/Li cells were tested with a cutoff voltage of 4.7-3.0 V. Li/Cu cells were assembled using 125 µm-thick Li foil. The separator was a glass micro-fiber disc, and the total electrolyte amount is 100 μL. Cell testing was conducted at 30°C. For the C-rate test, C/20 was used as charging current, and different currents were used for discharge for 3 cycles. Aurbach test^2^: 3 mAh/cm^2^ Li reservoir is plated on the Cu foil using 0.1 mA/cm^2^, then 0.6 mAh/cm^2^ Li was stripped/plated for 10 cycles before completely stripped to 1 V.

Contact angle measurements were conducted using Ramé-hart instrument (Model 90 Pro Edition) equipped with manual liquid dispensing system, U3 series super-speed digital camera (Fujifilm corporation, 1920 x 1080 resolution), and drop shape analysis software (DROPimage).

***X-ray photoelectron spectroscopy (XPS).*** XPS analysis was conducted on a PHI 5000 VersaProbe II system (Physical Electronics) with a base pressure of ∼2×10^-9^ torr. The spectra were obtained using an Al Kα radiation (hυ=1486.6 eV) beam (100 μm, 25 W), with Ar^+^ and electron beam sample neutralization, in Fixed Analyzer Transmission mode with a pass energy of 11.75 eV. Subtracting a Shirley background and then fitting the spectra to multiple Gaussian peaks was performed on all spectra using the Multipack software from Physical Electronics. The area under the XPS peaks (the sum of the Gaussian components) was adjusted using manufacturer-calibrated relative sensitivity factors and normalized to obtain elemental concentrations. The same normalization factors were used to plot XPS signal intensities as concentration per unit energy (at % eV^-1^). Binding energy was calibrated by shifting every region to align the C 1s peak of C-C/C-H environments at 284.8 eV.

***Scanning electron microscopy and transmission electron microscopy (SEM/TEM).*** The cycled coin cells were disassembled in the argon-filled glovebox, and the electrodes were thoroughly rinsed with anhydrous dimethyl carbonate and allowed to dry in glovebox. The morphologies and the elemental mapping of the cycled electrodes were examined using SEM JOEL JCM-6000-PLUS. TEM analysis was conducted in the JOEL JEM-2100F.

***Ex-situ X-ray diffraction (XRD).*** Ex-situ X-ray diffraction (XRD) patterns of the lithium plating with different electrolytes were collected with Bruker D-8 diffractometer. The recovered lithium metal electrodes after 5cycles of Li//Li symmetric test under the condition of 0.5mA/cm^2^ current density were sealed with Kapton protection layer to avoid unwanted air-exposure of lithium metal electrode in an Ar-filled glove box before the XRD measurement.

***Density functional theory methods****.* To understand the electronic properties of the fluorinated ethers, i.e., TTE, BTFE, and T_f_THF, density functional theory (DFT) calculations were carried out using Gaussian 09 simulation package^3^ with B3LYP functional^4^ and 6-31g basis set^5^.

Furthermore, to explore the electrolyte decomposition reaction mechanism on Li anode, mechanistic studies were carried out using plane-wave based Vienna Ab initio Simulation Package (VASP)^6^. The projector augmented wave (PAW)^7^ method was used to describe the wave function of the ionic cores along with the generalized gradient approximation (GGA) Perdew-Burk-Ernzerhof (PBE) functional^8^. For Li bulk optimization, the energy cutoff of 520 eV and 8 × 8 × 8 Monkhorst-Pack^9^ k-point meshes were used. Furthermore, periodic Li (110) surface was cleaved from the optimized bulk. For the geometry optimization over Li surface, the energy cutoff of 400 eV and 3 × 2 × 1 Monkhorst-Pack k-point meshes were used. The energy and geometries of the gas phase species were calculated by placing the molecule in a box with dimensions of 25 × 25 × 25 Å^3^. A single Γ-point was used for these calculations. For all VASP calculations, the convergence criterion for the self-consistent iteration is 1×10^-5^ eV; and the ionic relaxations stop when the force on each atom is less than 0.05 eV/Å.

***Molecular dynamics (MD) simulations.*** All MD simulations were performed on the Cray cluster Theta of the Leadership Computing Facility at Argonne National Laboratory. The simulations were carried out in a high-performance mode with version 2.14 of NAMD^10^, a greatly scalable molecular dynamic program used to render an atom-by-atom representation of biomolecules, created at the University of Illinois. All electrolyte simulations adopted GAMMP force field^11^ generated through GAAMP server on Laboratory Computing Resource Center at Argonne National Laboratory. The molar ratios of each molecular species, Li^+^, FSI^-^, P_f_Mpyr^+^ and diluents match the ratio in the experiments. The number of atoms for the simulated systems range between 81,830 and 100,000, and dimension of each simulation cell is ~100Å×100Å×100Å. All simulations adopt Periodic Boundary Condition under constant NVT condition. The equations of motion were integrated with a 2 fs time step, using Langevin dynamics at a temperature of 300 K. To overcome the slow dynamics due to the high viscosity of the simulated systems, Hamiltonian simulated annealing method (HSA)^12^ was employed to accelerate the equilibration of MD trajectories. In each HSA simulation, 64 independent trajectories were generated, and all of them were used to get statistical average of structural properties. Each trajectory lasts 20 ns and the last 10 ns was adopted to do statistical average. Snapshot of each trajectory was sampled at an interval of 100 ps, and thus for each simulated system, 6400 snapshots were adopted to guarantee high-fidelity sampling.

**Figure S1.** Cycling performance of NMC622/Li cell using LP_f_DME electrolyte with cutoff voltage of 4.7-3.0 V at 30 °C: (a) voltage profile of 1^st^ cycle charge and (b) discharge capacity and Coulombic efficiency.

**Figure S2.** Cyclic Voltammetry of 1 M LiFSI P_f_MpyrFSI scan between -0.5 to 6 V, using Pt/Li/Li as working/counter/reference electrodes with 10 mV/s scan rate.


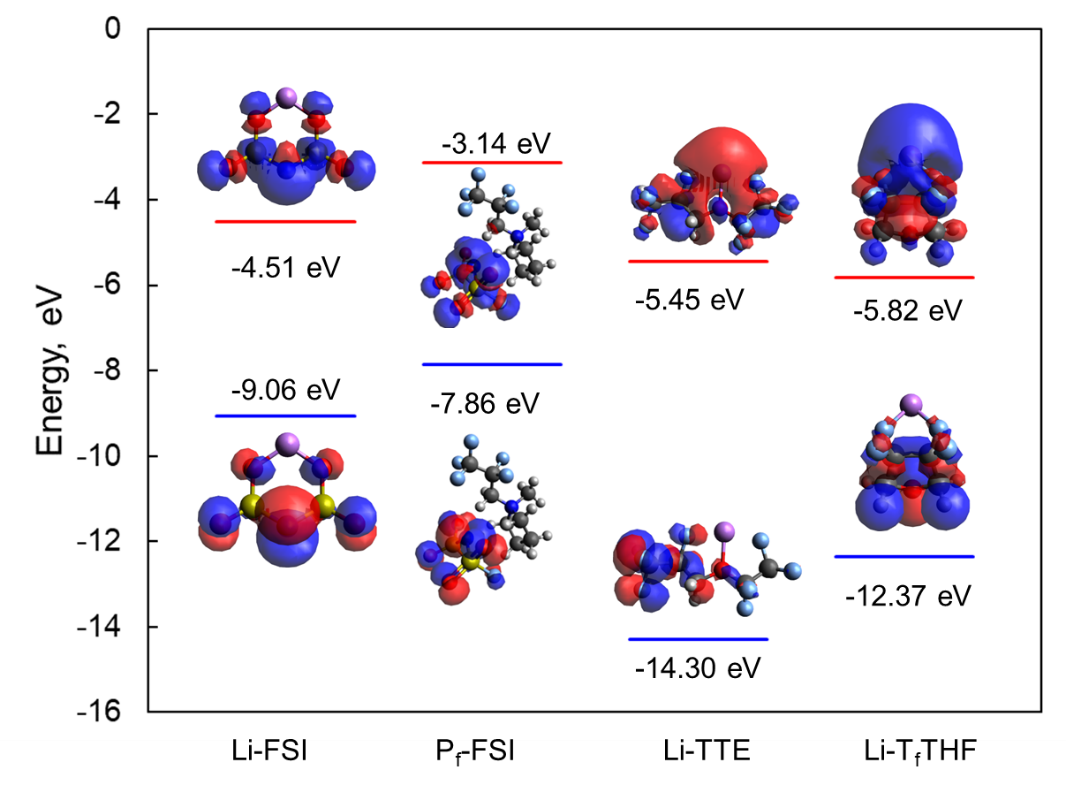


**Figure S3.** HOMO-LUMO energy levels of LiFSI_,_ P_f_MpyrFSI, Li^+^-TTE, and Li^+^-T_f_THF.

**Figure S4.** Contact angles of (a) LP_f_, (b) LP_f_TTE, and (c) LP_f_T_f_THF electrolytes on an Al foil substrate.

**Figure S5.** MD simulation on electrolyte structures. Radial distribution functions (RDFs) and coordination numbers P_f_Mpyr^+^ cation and diluents for (a) LP_f_, (b) LP_f_TTE, and (c) LP_f_T_f_THF electrolytes. (d) ^1^H NMR of LP_f_, LP_f_TTE, and LP_f_T_f_THF electrolytes with acetone-*d*_6_ as external reference.

**Figure S6.** Adsorption and decomposition energy on Li surface for (a) TTE, (b) T_f_THF.

**Figure S7.** Decomposition via deflourination (solid line) and deprotonation (dashed line) over Li surface for (a) TTE, (b) T_f_THF.

**Figure S8.** O 1s spectra from XPS analysis of recovered LiNiO_2_ cathodes using LP_f_, LP_f_TTE, and LP_f_T_f_THF electrolytes.

**Figure S9.** Ex-situ XRD of pristine and cycled Li metal harvested from Li/Li symmetric cells using Gen2, LP_f_, and LP_f_T_f_THF electrolytes.

**Reference**

1. Liu, Q.; Hsu, C.-W.; Dzwiniel, T. L.; Pupek, K. Z.; Zhang, Z., A Fluorine-Substituted Pyrrolidinium-Based Ionic Liquid for High-Voltage Li-Ion Batteries. *Chemical Communications* **2020,** *56* (53), 7317-7320.

2. Adams, B. D.; Zheng, J.; Ren, X.; Xu, W.; Zhang, J.-G., Accurate Determination of Coulombic Efficiency for Lithium Metal Anodes and Lithium Metal Batteries. *Adv. Energy Mater.* **2018,** *8* (7), 1702097.

3. Frisch, M. J.; Trucks, G. W.; Schlegel, H. B.; Scuseria, G. E.; Robb, M. A.; Cheeseman, J. R.; Scalmani, G.; Barone, V.; Petersson, G. A.; Nakatsuji, H.; Li, X.; Caricato, M.; Marenich, A. V.; Bloino, J.; Janesko, B. G.; Gomperts, R.; Mennucci, B.; Hratchian, H. P.; Ortiz, J. V.; Izmaylov, A. F.; Sonnenberg, J. L.; Williams; Ding, F.; Lipparini, F.; Egidi, F.; Goings, J.; Peng, B.; Petrone, A.; Henderson, T.; Ranasinghe, D.; Zakrzewski, V. G.; Gao, J.; Rega, N.; Zheng, G.; Liang, W.; Hada, M.; Ehara, M.; Toyota, K.; Fukuda, R.; Hasegawa, J.; Ishida, M.; Nakajima, T.; Honda, Y.; Kitao, O.; Nakai, H.; Vreven, T.; Throssell, K.; Montgomery Jr., J. A.; Peralta, J. E.; Ogliaro, F.; Bearpark, M. J.; Heyd, J. J.; Brothers, E. N.; Kudin, K. N.; Staroverov, V. N.; Keith, T. A.; Kobayashi, R.; Normand, J.; Raghavachari, K.; Rendell, A. P.; Burant, J. C.; Iyengar, S. S.; Tomasi, J.; Cossi, M.; Millam, J. M.; Klene, M.; Adamo, C.; Cammi, R.; Ochterski, J. W.; Martin, R. L.; Morokuma, K.; Farkas, O.; Foresman, J. B.; Fox, D. J. *Gaussian 16 Rev. C.01*, Wallingford, CT, 2016.

4. Becke, A. D., Density-Functional Thermochemistry .3. The Role of Exact Exchange. *J Chem Phys* **1993,** *98* (7), 5648-5652.

5. Hehre, W. J.; Ditchfield, R.; Pople, J. A., Self-Consistent Molecular-Orbital Methods .12. Further Extensions of Gaussian-Type Basis Sets for Use in Molecular-Orbital Studies of Organic-Molecules. *J Chem Phys* **1972,** *56* (5), 2257-+.

6. Kresse, G.; Furthmuller, J., Efficient Iterative Schemes for Ab Initio Total-Energy Calculations Using a Plane-Wave Basis Set. *Phys Rev B* **1996,** *54* (16), 11169-11186.

7. Blochl, P. E., Projector Augmented-Wave Method. *Phys Rev B* **1994,** *50* (24), 17953-17979.

8. Perdew, J. P.; Burke, K.; Ernzerhof, M., Generalized Gradient Approximation Made Simple. *Phys Rev Lett* **1996,** *77* (18), 3865-3868.

9. Monkhorst, H. J.; Pack, J. D., Special Points for Brillouin-Zone Integrations. *Phys Rev B* **1976,** *13* (12), 5188-5192.

10. Phillips, J. C.; Braun, R.; Wang, W.; Gumbart, J.; Tajkhorshid, E.; Villa, E.; Chipot, C.; Skeel, R. D.; Kalé, L.; Schulten, K., Scalable Molecular Dynamics with Namd. *J. Comput. Chem.* **2005,** *26* (16), 1781-1802.

11. Huang, L.; Roux, B., Automated Force Field Parameterization for Nonpolarizable and Polarizable Atomic Models Based on Ab Initio Target Data. *J. Chem. Theory Comput* **2013,** *9* (8), 3543-3556.

12. Jiang, W., Accelerating Convergence of Free Energy Computations with Hamiltonian Simulated Annealing of Solvent (Hsas). *J. Chem. Theory Comput* **2019,** *15* (4), 2179-2186.
